# Cost-Saving Early Diagnosis of Functional Pain in Nonmalignant Pain: A Noninferiority Study of Diagnostic Accuracy

**DOI:** 10.1155/2016/5964250

**Published:** 2016-03-21

**Authors:** Rafael J. A. Cámara, Christian Merz, Barbara Wegmann, Stefanie Stauber, Roland von Känel, Niklaus Egloff

**Affiliations:** ^1^University Medical Center of the Johannes Gutenberg University, Mainz, Institute of Medical Biostatistics, Epidemiology, and Informatics, Obere Zahlbacher Straße 69, 55131 Mainz, Germany; ^2^Department of General Internal Medicine, Division of Psychosomatic Medicine, Inselspital, Bern University Hospital, 3010 Bern, Switzerland; ^3^Heart Failure and Transplantation, Department of Cardiology, Inselspital, Bern University Hospital, 3010 Bern, Switzerland; ^4^Department of Psychosomatic Medicine, Clinic Barmelweid, 5017 Barmelweid, Switzerland; ^5^Department of Neurology, Inselspital, Bern University Hospital, 3010 Bern, Switzerland

## Abstract

*Objectives*. We compared two index screening tests for early diagnosis of functional pain: pressure pain measurement by electronic diagnostic equipment, which is accurate but too specialized for primary health care, versus peg testing, which is cost-saving and more easily manageable but of unknown sensitivity and specificity. Early distinction of functional (altered pain perception; nervous sensitization) from neuropathic or nociceptive pain improves pain management.* Methods*. Clinicians blinded for the index screening tests assessed the reference standard of this noninferiority diagnostic accuracy study, namely, comprehensive medical history taking with all previous findings and treatment outcomes. All consenting patients referred to a university hospital for nonmalignant musculoskeletal pain participated. The main analysis compared the receiver operating characteristic (ROC) curves of both index screening tests.* Results*. The area under the ROC curve for peg testing was not inferior to that of electronic equipment: it was at least 95% as large for finger measures (two-sided *p* = 0.038) and at least equally as large for ear measures (two-sided *p* = 0.003).* Conclusions*. Routine diagnostic testing by peg, which is accessible for general practitioners, is at least as accurate as specialized equipment. This may shorten time-to-treatment in general practices, thereby improving the prognosis and quality of life.

## 1. Introduction

The lifetime prevalence of chronic nonmalignant musculoskeletal pain varies between 13.5% and 47% in the general population [1]. In one-third of these patients, this pain is “functional,” which is defined as not explainable by lesions or inflammations of tissues or nerves [2, 3]. Because, as opposed to nociceptive and neuropathic pain, surgery and the analgesics scheme recommended by the World Health Organization are not adequate for functional pain, the distinction is clinically relevant (International Statistical Classification of Diseases and Related Health Problems: F45.40 and F45.41). Functional pain requires specialized multicomponent management involving physical exercise, activating physiotherapy, electrical, thermal, and tactile stimulation, relaxation techniques, psychological support, tricyclic antidepressants, and muscle relaxants [4]. Functional pain may occur as an isolated entity or as the dominant symptom of “central sensitivity syndromes” such as fibromyalgia [5, 6], whereby the functional alteration of the nervous system is probably involved [7, 8].

A delay of eight years on average to classify pain as either functional, nociceptive, or neuropathic increases expenditures and reduces the prognosis and the quality of life of the patients [3, 4, 9, 10]. Because imaging and laboratory findings often seem to correspond to the symptoms at the first evaluation, diagnosing functional pain by the exclusion of nociceptive and neuropathic pain is difficult [3, 8]. When pain is functional, namely, prolonged by central nervous system sensitization [5, 7], nociceptive stimuli such as whiplash-associated pain syndrome or osteoarthritis and inflammation are often mistaken for its cause [3, 11–13]. The diagnostic process involves repetitions of, for instance, computer tomographies, electromyographies, infiltrations of local anesthetics, surgery, and extensive laboratory examinations [3].

Despite the importance of distinguishing functional from nociceptive and neuropathic pain early, screening tests are still missing in primary care [4, 11–13]. Techniques to measure pressure pain sensitivity, summarized as electronic algometry, are very precise (one kilopascal), but expensive, and thus only used in specialized centers and studies [14–17]. They show that the pain threshold is reduced in many functional pain syndromes [5–7]. While electronic algometry is indispensable for research, cost-effective and more easily manageable alternatives are required for functional pain screening in primary care [18].

Our hypothesis was that peg algometry's ability to distinguish functional from nociceptive and neuropathic pain is not inferior to that of electronic algometry, which has been widely investigated but is many times more costly than peg algometry and only used by specialists. The latter is a recently introduced test performed with a pressure pain device that easily fits into a coat pocket. We had shown that peg algometry is very reliable and consistent according to criteria of the British Standards Institution [19, 20]. For instance, standardized Bland Altman repeatability coefficients were 0.96 for finger peg algometry and even 0.63 for ear peg algometry. Moreover, 95% of the differences between test and retest results were within two standard deviations from “no difference” for ear lobe, and even within one standard deviation for middle finger peg algometry, with all *p* values <0.001.

## 2. Methods

The enrolment and data collection for this noninferiority study of diagnostic accuracy comparing the two index tests, namely, peg with electronic algometry, were carried out at the Clinical Departments for Psychosomatic Medicine and for Orthopedics at the Bern University Hospital. The local ethics committee had approved the study protocol. All eligible patients were informed about the study. The main eligibility criteria were hospitalization for nonmalignant musculoskeletal pain and written informed consent. Further eligibility criteria were the absence of local lesions at the site of measurement or at an efferent innervation of this site.

Following eligibility assessment, an advanced medical student read the index tests, and the attending specialists, who ignored the readings of the index tests, assessed the reference standard. All passed their written results to the data manager. The enrolment continued until at least 60 patients with functional and 60 patients with nociceptive or neuropathic pain consented (see “analysis and presentation of data”). The STARD checklist for reporting studies on diagnostic accuracy [21] was followed for reporting the introduction, methods, and results part (see Supplementary Material 1 available online at http://dx.doi.org/10.1155/2016/5964250), and the STROBE checklist [22] was used for the discussion.

The reference standard for comparing the two index tests was a thorough review of the medical history including clinical, imaging, arthroscopic, and surgical findings and treatment outcomes according to the criteria of the International Statistical Classification of Diseases and Related Health Problems. By reviewing pain histories of eight years on average, exclusion of alternate pain causes and little effect of conventional analgesics confirmed* functional* pain [23]. The correlation of the localization and character of pain symptomatology with clinical and investigative findings from the patients' histories corroborated* nociceptive* or* neuropathic* pain. Functional pain was found, for instance, in somatoform pain, fibromyalgia, complex regional pain syndrome, chronic temporomandibular joint disorder, whiplash disorder, and chronic low back pain, as well as combinations of such disorders. However, neither the given diagnosis nor any central nervous system hypersensitivity testing was our reference standard, but rather a long and costly exclusion of lesions, degenerations, and inflammations of tissues or nerves which could have explained the pain, as well as failure of conventional pain treatment [2, 3].

Combinations of functional and nociceptive pain were classified as functional pain. Such combinations are frequent [24], and early diagnosis of the functional pain component is as important in such combinations as in isolated functional pain. Missing neither the one nor the other remains a challenge. Having said that, the focus of this study was the identification of patients requiring multicomponent pain treatment, from which most patients with functional pain might benefit, whether or not they also have nociceptive pain.

Standardized pegs of polypropylene were used for peg algometry [20], and a device that had been tested in multiple studies and is regularly employed in pain research was used for electronic algometry according to the manufacturer's instructions [25]. Whether to start with peg or with electronic algometry was determined by flipping a coin. Both algometry techniques were applied to the distal phalanx of the middle finger and then to the ear lobe. Measurements were performed at the left and the right side of the body in order to control for side-specific differences of perception [26]. A pause of one minute before each measurement aimed to minimize sensitization phenomena. Finger algometry was conducted without touching the nail fold, and ear algometry was performed in the middle of the ear lobe without touching cartilaginous structures. All algometry results were immediately documented for later calculation of the mean between the left and the right side.

The peg was left in place for 10 seconds. Before removing it, the study participants rated their pain sensitivity numerically on a scale from 0 to 10 with intervals of 0.5, where “0” was “no pain at all” and “10” was “the worst imaginable pain.” Each of the four anatomical sites (left and right middle finger as well as left and right ear lobe) was measured once.

In contrast to peg algometry, electronic algometry increased the pressure at 50 kilopascals per second until the study participants experienced the pressure as pain. At that moment, they pressed a button to display the pressure pain threshold in kilopascals. To proceed as recommended by the manufacturer, electronic algometry was conducted three times for each of the anatomical sites (see the previous paragraph), and the average of the three readings was computed before computing the average between the left and right sides.

In order to address potential interviewer-bias and competing interests, the reader of the index tests knew that the internal consistency of peg and electronic algometry would be investigated but ignored the reference standard and the diagnostic accuracy comparison [20]. She was involved neither in the assessment of the medical history of the study participants nor in the management of their pain. Once she had forwarded the results of peg and electronic algometry to the data manager, she was granted access to the participants' files to extract descriptive data that she sent to the data manager. To improve consistency, the index tests were performed by the same person and in the same manner across all study participants. To relax the participants, the measurements were performed in their rooms.

The areas under the receiver operating characteristic curves (AUC) enabled a direct comparison of both algometry techniques (roc, Stata SE®, version 12.1). The larger an AUC, the higher the probability of detecting functional pain if there is any and of excluding it if there is none. An AUC of 50% would correspond to an uninformative test, and an AUC of 100% to a perfectly discerning test. Graphs were drawn for visual comparison (roccomp, Stata SE, version 12.1). Statistical significance was tested by noninferiority comparisons. To compute the standard error of the difference between the AUCs of the index tests, the correction factor for the correlation between these two techniques was needed, which was computed by the functional-pain-nonfunctional-pain average of their correlation coefficients (ktau, Stata SE, version 12.1) and the average of their AUCs (roctab, Stata/SE, version 12.1) [27].

Noninferiority of peg to electronic algometry was assumed if the AUC of peg algometry was at least 80% as large as the AUC of electronic algometry against a *p* value smaller than 0.05 [20].

The reference variable was “functional versus nociceptive and neuropathic pain,” and the two classification variables to compare were “numerical pain rating of peg algometry” and “pain threshold of electronic algometry in kilopascals.”

The aim was to detect noninferiority of peg to electronic algometry, as defined above, with 80% power. Assuming a rank correlation of peg and electronic algometry of 0.4 [20] and an AUC of electronic algometry of 80% [15, 16], a minimum of 60 study participants with functional pain and 60 study participants with nociceptive pain were required for this aim [27].

The study participants were described using Stata/SE, version 12.1. The description included “peg algometry rating from 0 to 10 in 1/2 units,” “electronic algometry in kilopascals,” “age, sex, pain onset, and pain localization,” “numeric analogue scales from 0 to 10 in 1/2 units,” and medication. “Numeric analogue scales from 0 to 10 in 1/2 units” included musculoskeletal pain and mood. Medication included “analgesics,” “antidepressants (yes/no),” and “antiepileptic drugs (yes/no).” Analgesics were categorized as “strong opiates,” “weak opiates,” “nonopiate analgesics only,” and “no analgesics.” A table describes these variables as counts and percentages if they are binary or categorical and other types of variables as means and standard deviations. It also provides the ranges of the index tests for the identification of outliers.

## 3. Results

**Figure 1:**
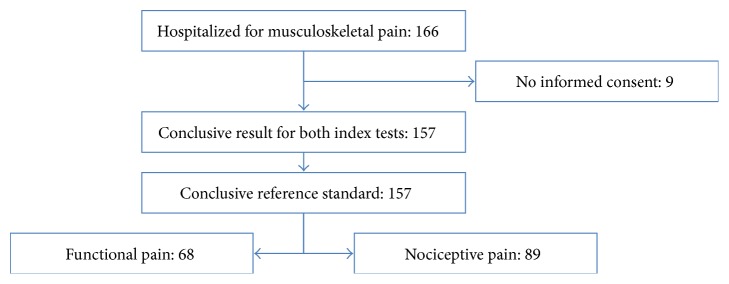
Flow of a referred sample of 166 individuals hospitalized for musculoskeletal pain. The reference standard, namely, a thorough review of the clinical pain history including past investigations and pharmacological responses, revealed 68 participants with functional and 89 participants with nociceptive pain.

**Figure 2:**
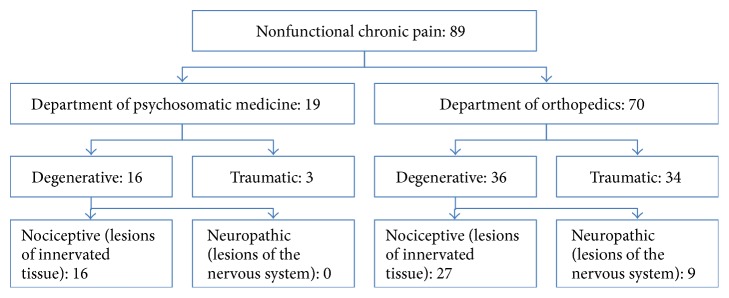
Classification of nonfunctional pain according to study site and pathology. Nine individuals of the nociceptive pain group of 89 participants had neuropathic rather than nociceptive pain. This number was too small for sensitivity analyses.

Of 166 individuals hospitalized for musculoskeletal pain from May to August 2009 and January to March 2010, 157 consented to participate, of which none had to be excluded (Figure 1). The nociceptive pain group of 89 participants included nine patients with neuropathic rather than nociceptive pain (Figure 2). Due to the delayed process until referral to specialized therapeutic centers [3], study participants with this condition had experienced pain for a longer period of time and differed in medications (Table 1).

**Figure 3:**
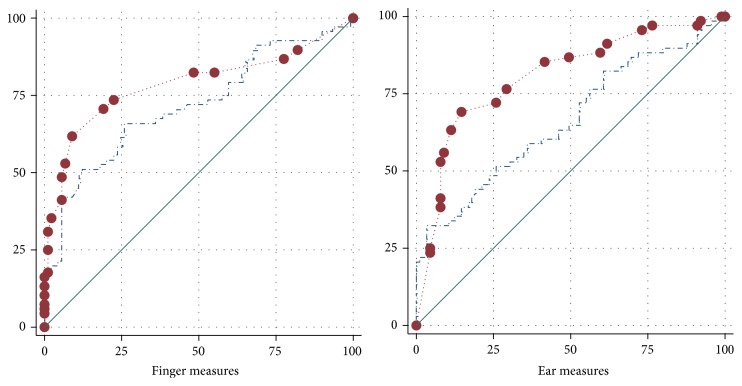
Visual comparison of peg and electronic algometry in discerning functional from nociceptive pain: receiver operating characteristic curves. Axes: *x*-axis = 100%  −  specificity in %; *y*-axis = sensitivity in %. Peg algometry: the red circles reflect the maximum of 21 possible test results given by the 0 to 10 pain rating at intervals of 0.5 points. Electronic algometry: the blue dashed line reflects the multitude of possible test results provided by the pressure pain measurement unit “kilopascal” with standard deviations ranging between 72 and 97 kilopascals.

The visual comparison showed that, for finger and ear measures, the AUCs for peg algometry were larger than those for electronic algometry (Figure 3).

The noninferiority comparison in Table 2 showed that, for finger measures, the AUC for peg algometry was at least 95% as large as that for electronic algometry (*p* = 0.038), and for ear measures, the AUC for peg algometry was at least equally as large as that for electronic algometry (*p* = 0.003).

## 4. Discussion

Taken together, we found that finger peg algometry is at least 95% as reliable as finger electronic algometry in distinguishing functional from nociceptive and neuropathic musculoskeletal pain, and we found that ear peg algometry is even equally good or better than ear electronic algometry. Cost-effective and more easily manageable alternatives for electronic algometry were developed in the past [18], but we were the first to investigate their accuracy to differentiate functional from nociceptive musculoskeletal pain. One strength of this study is that we used identical conditions to compare a new screening test accessible for primary care with an established research test of known accuracy [14–17]. This means that most potential limitations would affect the absolute performance of both tests, thereby leaving the comparison between them only minimally affected, it at all.

A pain history of unsuccessful examinations for and treatment of assumed organic pain-sustaining factors is a strong reference standard because it has very important clinical implications for the patients [3, 4]. However, this reference standard might also include the limitation of unequal years of pain history and medication between study participants with functional and nociceptive pain, as well as corroboration of the reference standard in a sample referred to specialized centers rather than in general practices. In addition, we cannot exclude that the patients spoke about their pain history to the person who performed the index tests. However, this person ignored the study objectives and had no competing interests. In addition, the pressure algometry depends on the perception of the patients but not on the perception of the tester. Prior to the results of this study, there was no good reference standard for an early pain stage [11–13], and only at an early pain stage there are patients certainly blind to their condition who do not differ substantially in terms of medication.

Since we found that an economical device is at least as good at screening for functional musculoskeletal pain as a tried and trusted device used in specialized departments, screening is also possible in primary care. This does not mean that peg algometry provides diagnostic certitude at the very onset but that it is a useful screening tool. At onset, musculoskeletal pain might result from altered function of the nervous system (functional pain) and from nociceptive or neuropathic stimuli such as injury, inflammation, or degeneration. In the event of suspected functional pain, this would make a parallel investigation of different causes mandatory [3, 7]. But as pain starts to become chronic [1], having screened for central nervous system sensitization may support general practitioners in determining whether an injury or a disease might have been overseen or inadequately treated, or whether it is more likely that the pain is functional [3, 5, 7].

It would be interesting to know how well peg algometry carried out before initiation of extensive diagnostic and therapeutic procedures predicts their success. Since we obtained our findings in a highly specialized setting, caution is appropriate when generalizing them to primary care. However, a gain in certitude regarding generalizability would require a long observation period in a primary care setting for which the expensive electronic algometry is not accessible. Then again, we found that peg algometry is at least as good as electronic algometry. We deduce that the research we suggest is possible only with peg algometry. In an upcoming third phase, we plan to investigate the difference in therapeutic success between taking into account and ignoring the screening results of peg algometry.

What we can say now is that general practitioners can start appropriate pain treatment earlier, because early separation of functional from nociceptive and neuropathic pain becomes easier. Interested general practitioners may manufacture their own peg algometers, which is also possible in countries with few resources. They may explain to their patients that a short innocuous test performed instantly with a small device of familiar appearance can help to tell if there is probably no alteration of the painful tissue behind the very frequent condition of musculoskeletal pain. If the test unmasks an enhanced reactivity of the central nervous system to pain stimuli (central nervous system sensitization) [5, 7], patients may prefer knowing this early rather than hearing that “nothing” has been found. Awareness that their central nervous system is even likely to continue reacting after removal of the initial pain stimulus might reduce insecurity if the pain becomes chronic [9]. Even if patients have a combination of functional and nociceptive pain, unmasking a hypersensitivity component might help understanding why the standard treatment remains unsatisfactory and additional treatment modalities are required.

As appropriate pain treatment can start earlier, the prognoses of functional, nociceptive, and perhaps even neuropathic pain are likely to improve [3, 4]. This might be perceived with more personal satisfaction than years of diagnostic and therapeutic incertitude [3, 9].

## Supplementary Material

The completed STARD checklist shows which pages of the article address which items for reporting diagnostic accuracy studies (, last access on February 2016).

## Figures and Tables

**Table 1:** Cross-sectional description of study participants with functional and nociceptive pain^a^.

	Functional pain	Nociceptive pain
Number of participants (denominator)	68	89
Peg algometry rating from 0 to 10 in 1/2 units		
Mean finger pressure pain (SD)	4.5 (3.0)	1.5 (1.5)
Mean ear lobe pressure pain (SD)	7.5 (2.5)	4.5 (2.5)
Range of finger pressure pain^b^	0 to 10	0 to 7
Range of ear lobe pressure pain^b^	1 to 10	0 to 10
Electronic algometry in kilopascals		
Mean finger pain threshold (SD)	156 (96)	228 (97)
Mean ear lobe pain threshold (SD)	125 (85)	164 (72)
Range of finger pain threshold^b^	12 to 467	71 to 676
Range of ear lobe pain threshold^b^	2 to 461	47 to 368
Age, sex, pain onset, and pain localization		
Mean age in years (SD)	46 (12)	57 (16)
Number of women (%)	40 (59)	37 (42)
Mean pain duration in months (SD)	98 (114)	35 (75)
Number of pain onsets before the 50th birthday (%)	59 (87)	40 (45)
Number of patients with multilocular pain (%)	56 (82)	33 (37)
Numeric analogue scales from 0 to 10 in 1/2 units		
Mean intensity of musculoskeletal pain (SD)	6.5 (2.0)	4.0 (2.0)
Mean mood (SD)	6.0 (2.5)	4.0 (2.0)
Medication		
Number of patients		
With strong opiates (%)	10 (15)	14 (16)
With weak opiates (%)^c^	12 (18)	6 (7)
With nonopiate analgesics only (%)	33 (49)	67 (75)
Without analgesics (%)	13 (19)	2 (2)
Number of patients with antidepressants (%)	58 (85)	17 (19)
Number of patients with antiepileptic drugs (%)	16 (24)	10 (11)

^a^No missing data; ^b^no outliers; ^c^weak opiates = tramadol and codeine.

**Table 2:** Noninferiority comparison of peg and electronic algometry in discerning functional from nociceptive pain: areas under the receiver operating characteristic curves (AUC)^a^.

	AUC in % of a perfectly discerning test (95% CI)		Difference (95% CI) *p* value	
	Peg algometry	Electronic algometry	Crude	AUC of peg minus 95% of the AUC of electronic algometry
Finger measures	79 (71 to 86)	72 (64 to 80)	7 (−3 to 16) 0.18	11 (1 to 20) 0.04
Ear measures	81 (74 to 88)	66 (57 to 75)	15 (5 to 24) 0.003	18 (8 to 27) < 0.001

^a^No missing data.

## References

[1] Cimmino M. A., Ferrone C., Cutolo M. (2011). Epidemiology of chronic musculoskeletal pain. *Best Practice & Research Clinical Rheumatology*.

[2] Jackson J. L., Passamonti M. (2005). The outcomes among patients presenting in primary care with a physical symptom at 5 years. *Journal of General Internal Medicine*.

[3] Atzeni F., Cazzola M., Benucci M., Di Franco M., Salaffi F., Sarzi-Puttini P. (2011). Chronic widespread pain in the spectrum of rheumatological diseases. *Best Practice and Research: Clinical Rheumatology*.

[4] Turk D. C., Wilson H. D., Cahana A. (2011). Treatment of chronic non-cancer pain. *The Lancet*.

[5] Yunus M. B. (2007). Fibromyalgia and overlapping disorders: the unifying concept of central sensitivity syndromes. *Seminars in Arthritis and Rheumatism*.

[6] Egloff N., Cámara R. J., von Känel R., Klingler N., Marti E., Ferrari M.-L. (2014). Hypersensitivity and hyperalgesia in somatoform pain disorders. *General Hospital Psychiatry*.

[7] Basbaum A. I., Bautista D. M., Scherrer G., Julius D. (2009). Cellular and molecular mechanisms of pain. *Cell*.

[8] Gatchel R. J., Peng Y. B., Peters M. L., Fuchs P. N., Turk D. C. (2007). The biopsychosocial approach to chronic pain: scientific advances and future directions. *Psychological Bulletin*.

[9] Nettleton S., Watt I., O'Malley L., Duffey P. (2005). Understanding the narratives of people who live with medically unexplained illness. *Patient Education and Counseling*.

[10] Annemans L., Wessely S., Spaepen E. (2008). Health economic consequences related to the diagnosis of fibromyalgia syndrome. *Arthritis & Rheumatism*.

[11] Fitzcharles M.-A., Boulos P. (2003). Inaccuracy in the diagnosis of fibromyalgia syndrome: analysis of referrals. *Rheumatology*.

[12] Klaus K., Rief W., Brähler E., Martin A., Glaesmer H., Mewes R. (2013). The distinction between ‘medically unexplained’ and ‘medically explained’ in the context of somatoform disorders. *International Journal of Behavioral Medicine*.

[13] Dagfinrud H., Storheim K., Magnussen L. H. (2013). The predictive validity of the Örebro Musculoskeletal Pain Questionnaire and the clinicians' prognostic assessment following manual therapy treatment of patients with LBP and neck pain. *Manual Therapy*.

[14] Arendt-Nielsen L., Yarnitsky D. (2009). Experimental and clinical applications of quantitative sensory testing applied to skin, muscles and viscera. *Journal of Pain*.

[15] Tastekin N., Uzunca K., Sut N., Birtane M., Mercimek O. B. (2010). Discriminative value of tender points in fibromyalgia syndrome. *Pain Medicine*.

[16] Scudds R. A., Rollman G. B., Harth M., McCain G. A. (1987). Pain perception and personality measures as discriminators in the classification of fibrositis. *Journal of Rheumatology*.

[17] Neziri A. Y., Curatolo M., Limacher A. (2012). Ranking of parameters of pain hypersensitivity according to their discriminative ability in chronic low back pain. *Pain*.

[18] Johnson T. W., Watson P. J. (1997). An inexpensive, self-assembly pressure algometer. *Anaesthesia*.

[19] British Standards Institution (1998). Accuracy (trueness and precision) of measurement methods and results—part 5: alternative methods for the determination of the precision of a standard measurement method. *BS ISO*.

[20] Egloff N., Klingler N., von Känel R. (2011). Algometry with a clothes peg compared to an electronic pressure algometer: a randomized cross-sectional study in pain patients. *BMC Musculoskeletal Disorders*.

[21] Bossuyt P. M., Reitsma J. B. (2003). Standards for reporting of diagnostic accuracy. The STARD initiative. *The Lancet*.

[22] Von Elm E., Altman D. G., Egger M., Pocock S. J., Gøtzsche P. C., Vandenbroucke J. P. (2007). The strengthening the reporting of observational studies in epidemiology (STROBE) statement: guidelines for reporting observational studies. *The Lancet*.

[23] Schuepbach W. M., Adler R. H., Sabbioni M. E. (2002). Accuracy of the clinical diagnosis of ‘psychogenic disorders’ in the presence of physical symptoms suggesting a general medical condition: a 5-year follow-up in 162 patients. *Psychotherapy and Psychosomatics*.

[24] Wolfe F., Clauw D. J., Fitzcharles M.-A. (2011). Fibromyalgia criteria and severity scales for clinical and epidemiological studies: a modification of the ACR preliminary diagnostic criteria for fibromyalgia. *The Journal of Rheumatology*.

[25] http://www.somedicprod.se/uk/algometer-uk.shtml.

[26] Egloff N., Maecker F., Stauber S., Sabbioni M. E., Tunklova L., von Känel R. (2012). Nondermatomal somatosensory deficits in chronic pain patients: are they really hysterical?. *Pain*.

[27] Hanley J. A., McNeil B. J. (1983). A method of comparing the areas under receiver operating characteristic curves derived from the same cases. *Radiology*.

